# Assessment of the efficacy of various maxillary molar intrusion therapies: a systematic review

**DOI:** 10.1186/s40510-023-00490-3

**Published:** 2023-11-13

**Authors:** Sarah Abu Arqub, Dalya Al-Moghrabi, Marissa G. Iverson, Philippe Farha, Hala Abdullah Alsalman, Flavio Uribe

**Affiliations:** 1 Division of Orthodontics, University of Florida, Gainesville, FL USA; 2 Department of Preventive Dental Sciences, College of Dentistry, Princess Nourah bint Abdulrahman University, Riyadh, Saudi Arabia; 3 L.M. Stowe Library, University of Connecticut Health, Farmington, CT USA; 4 Boston University Henry M. Goldman School of Dental Medicine, Boston, MA USA; 5 Division of Orthodontics, Department of Craniofacial Sciences, University of Connecticut Health, Farmington, CT USA

**Keywords:** Maxillary molar intrusion, Temporary anchorage devices, Posterior bite blocks, Open bite bionator, Rapid molar intruder

## Abstract

**Aims:**

To systematically assess the efficacy of the various interventions used to intrude maxillary molars. Furthermore, to evaluate associated root resorption, stability of intrusion, subsequent vertical movement of mandibular molars, cost effectiveness, compliance, patient reported outcomes and adverse events.

**Methods:**

A pre-registered and comprehensive literature search of published and unpublished trials until March 22nd 2023 with no language restriction applied in PubMed/Medline, Embase, Scopus, DOSS, CENTRAL, CINAHL Plus with Full Text, Web of Science, Global Index Medicus, Dissertation and Theses Global, ClinicalTrials.gov, and Trip (PROSPERO: CRD42022310562). Randomized controlled trials involving a comparative assessment of treatment modalities used to intrude maxillary molars were included. Pre-piloted data extraction forms were used. The Cochrane Risk of Bias tool was used for risk of bias assessment, and The Grading of Recommendations Assessment, Development, and Evaluation (GRADE) system was used for certainty of evidence appraisal.

**Results:**

A total of 3986 records were identified through the electronic data search, of which 24 reports were sought for retrieval. Of these, 7 trials were included. One trial was judged at high risk of bias, while the others had some concerns. Based on individual small sample studies, maxillary molar intrusion was achieved using temporary anchorage devices (TADs) and rapid molar intruder appliance (RMI). It was also observed to a lesser extent with the use of open bite bionator (OBB) and posterior bite blocks. The molar intruder appliance and the posterior bite blocks (spring-loaded or magnetic) also intruded the lower molars. Root resorption was reported in two studies involving TADs. None of the identified studies involved a comparison of conventional and TAD-based treatments for intrusion of molars. No studies reported outcomes concerning stability, cost-effectiveness, compliance and patient-reported outcomes. Insufficient homogeneity between the included trials precluded quantitative synthesis. The level of evidence was very low.

**Conclusions:**

Maxillary molar intrusion can be attained with different appliances (removable and fixed) and with the use of temporary anchorage devices. Posterior bite blocks (spring-loaded or magnetic) and the RMI offer the additional advantage of intruding the mandibular molars. However, stability of the achieved maxillary molar intrusion long term is unclear. Further high-quality randomized controlled trials are needed.

## Background

Intrusion is one of the most mechanically challenging types of tooth movement. It has been described as the apical movement of the geometric center of the root in respect to a plane perpendicular to the long axis of the tooth [1]. The mechanical stresses are often increased with intrusion at the root apex, which might increase the risk of root resorption with this specific type of tooth movement [2].

With regard to intruding posterior teeth, molar intrusion is a treatment option for patients with Anterior Open Bite (AOB); a malocclusion often characterized by the overeruption of the posterior teeth or/and under eruption of the anterior teeth [3]. Furthermore, unopposed maxillary molars for a prolonged period tend to supraerupt and encroach on the space of its lower counterpart. It has been reported that 82% of subjects presented with supraerupted maxillary molars would require adjunctive orthodontic restorative and/or endodontic interventions prior to prosthetic replacement for the opposing teeth to correct interocclusal space deficiency [4]. Therefore, orthodontic intrusion is a clinically desired treatment option for supraerupted teeth.

Many treatment modalities have been suggested to control the vertical dimension in different age groups of treated subjects. These include conventional methods such as utilization of high pull headgear [5], functional appliances [6], vertical-pull chin cups [7], active vertical correctors [8], and posterior bite blocks with either springs or magnets [9]. Besides the fact that most of the above-mentioned modalities demand high level of patient cooperation, their clinical success was often correlated with the younger age group [6]. Moreover, achieving predictable intrusive movement in non-growing subjects is considered an onerous task, and depending on the intrusion severity it might involve endodontic treatment and coronal reduction or extraction [10]. Moreover, magnetic bite blocks are associated with poor three-dimensional control due the devices being deviated from the centered relation contact [9].

The introduction of temporary anchorage devices (TADs) has facilitated performance of challenging tooth movements more predictably by eliminating the compliance factor and providing absolute anchorage while intruding teeth, furthermore, preventing side effects on adjacent teeth during treatment [11]. However, TADs are not often prescribed for young individuals with deciduous or early mixed dentition [12], therefore alternative mechanisms for intrusion should be considered in this age group. Nevertheless, despite their reported failure rate, the use of TADs ensures the delivery of uninterrupted and continuous forces during intrusion.

Overall, molar intrusion even with all proposed treatment mechanisms remains difficult and unpredictable, and multiple factors play a role in the success of intruding posterior teeth, such as patients’ compliance, age, bone density, number of teeth intruded, severity of malocclusion, appliance used and site of force application when using TADs (palatal, buccal or both) [13, 14]. Moreover, molars are large multirooted teeth and the mandibular cortex is dense, therefore, intruding mandibular teeth is often more challenging than intruding the maxillary teeth [15]. Reported complications of molar intrusion with the use of TADs include relapse [16] as well as root resorption [17].

Previous systematic reviews primarily investigated clinical effects of molar intrusion for correction of open bite cases [6, 16, 18, 19]. Only one previous review published in 2006 attempted to quantify the true amount of intrusion achieved during orthodontic treatment [20]. Therefore, identification of the expected amount and rate of intrusion with various appliances would be of marked value to clinicians. This systematic review aimed to critically evaluate and compare the efficacy of the various interventions used to intrude maxillary molars. Furthermore, this review sets out to appraise outcomes related to intrusion including root resorption, stability, subsequent vertical movement of mandibular molars, cost effectiveness, patient reported outcomes and adverse events that might accompany the use of various appliances for intrusion.

## Materials and methods

### Protocol and registration

The systematic review protocol was registered prior to commencement in the National Institute of Health Research’s PROSPERO Protocol Registry (https://www.crd.york.ac.uk/prospero/; trial registration number: PROSPERO CRD42022310562). This systematic review was conducted in accordance with the Cochrane Handbook for systematic reviews and interventions [21] and the guidelines of Preferred Reporting Items for Systematic Reviews and Meta-Analyses (PRISMA) [22].

### Eligibility criteria

The PICOS scheme for this systematic review is presented in Table 1. Only randomized controlled trials reporting baseline and outcome data related to the amount of maxillary molar intrusion using conventional treatment modalities and/or with the use of temporary anchorage devices (TADs). Primary outcomes included amount and rate of maxillary molar intrusion, and secondary outcomes included amount of root resorption, stability of intrusion, patient reported outcomes, compliance, cost effectiveness, adverse effects and vertical movement of mandibular molars. No restriction on language or date of publication were applied.

**Table 1:** Eligibility criteria for the present systematic review

Domain	Inclusion criteria	Exclusion criteria
Participants	Orthodontic patients requiring maxillary molar intrusion for either open bite correction or pre-prosthetic applications, without age or gender predilection	Participants with systemic diseases or disabilities Subjects treated with orthognathic surgery or surgical adjunctive procedures Subjects in primary dentition stage
Interventions	Intrusion of maxillary molars with conventional treatment modalities (posterior bite blocks, headgear, magnets, vertical chin cup, spring loaded bite blocks, removable or fixed appliances) and/or with the use of TADs	Surgically assisted treatment protocols for posterior segment intrusion
Comparison	Intrusion of maxillary molars with conventional or TAD based mechanisms, or control group	
Outcomes	*Primary outcomes* Amount of maxillary molar intrusion Rate of maxillary molar intrusion *Secondary outcomes* Amount of root resorption associated with intrusion Vertical movement of mandibular molars Stability of intrusion Patient reported outcomes Compliance Cost effectiveness Adverse effects No restrictions on data collection sources (dental models, clinical measurements and radiographs were considered)	
Study design	Randomized controlled trials	Animal studies, prospective non-randomized controlled trials, retrospective studies, systematic reviews, case reports and case series

TAD: temporary anchorage device

#### Search strategy

The following databases and trials registries were searched from date of inception to March 22nd, 2023 (Appendix 1): PubMed/MEDLINE (including Pre-MEDLINE and non-MEDLINE; 1945 to March 2023), Embase (Elsevier; 1947 to March 2023), Scopus (Elsevier; 1966 to March 2023), Dentistry & Oral Science Source (DOSS, Ebsco; 1919 to March 2023), Cochrane Central Register of Controlled Trials (CENTRAL; Wiley; through March 2023), CINAHL Plus with Full Text (Ebsco; 1937 to March 2023), Web of Science (Medline, Biosis, and the Zoological Record only; Clarivate; 1895 to March 2023), Global Index Medicus (World Health Organization; 1917 to March 2023), Dissertations & Theses Global (ProQuest; 1861 to March 2023), ClinicalTrials.gov (through March 2023), and the Trip Database (Trip Database Ltd, tripdatabase.com; through March 2023; Appendix 1). That was developed by an experienced health sciences librarian and the authors (AS, AD, IM). The cited references and citing references of all included studies were screened.

#### Studies selection and data extraction

Two authors (AS, FP) screened the retrieved records for eligibility assessment. Selection was based on title, abstract, study design, and full text reading if needed, to match the inclusion criteria. Conflicts were resolved by contacting a third author (AD).

Using a customized data collection form, data extraction was carried out by two reviewers (AS, AH) independently and in duplicate. Conflicts were resolved by discussion with a third author (AD). Extracted data included: type of study, clinical setting, sample size, demographic information, type of appliance, details about the intervention, intrusion period, force levels, prescribed wear time for removable appliances, retention protocol if mentioned, outcome measures (primary: amount and rate of maxillary molar intrusion; secondary: subsequent vertical movement of lower molars, root resorption, stability, compliance, cost effectiveness, patient reported outcomes if any, and adverse events related to failure rates or reported harms).

#### Risk of bias in individual studies

The risk of bias for the included randomized controlled trials was assessed using the Cochrane’s tool (RoB 2) [23] by two reviewers, independently (AS, AD). Any disagreement was resolved by a third author (UF). Studies were deemed to be of high risk of bias (if at least one domain was rated at high risk), some concerns (if at least one domain was classified at unclear risk of bias) and low (if majority of domains present with low risk of bias).

#### Quality of evidence

Quality of evidence was judged according to the Grading of Recommendations, Assessment, Development, and Evaluation (GRADE) approach [24]. It was used to appraise the overall quality of evidence of the primary outcomes (the amount and rate of intrusion of the maxillary molars) reported in the included studies. Evidence was judged as very low, low, moderate, and high based on 5 domains, risk of bias, precision, consistency, directness, and other aspects outlined in the included studies. Evaluation was conducted independently by two authors (AS, AD) and in duplicate.

#### Risk of bias across studies

Standard and contoured enhanced funnel plots were planned if sufficient number of trials (more than 10 trials) were included in the meta-analysis if executed.

#### Dealing with missing data

In case of any missing information, corresponding authors of the included trials were contacted.

#### Data synthesis

It was preplanned to perform a meta-analysis, only if sufficient data and adequate studies were available to justify a valid statistical analysis. Clinical heterogeneity of studies was determined by assessing the study characteristics with particular emphasis on characteristics of participants, types of interventions, and outcomes. Weighted treatment effect with its 95% confidence intervals was to be calculated. *I*^2^ test was to be used to quantify statistical heterogeneity.

## Results

### Study selection

**Fig. 1:**
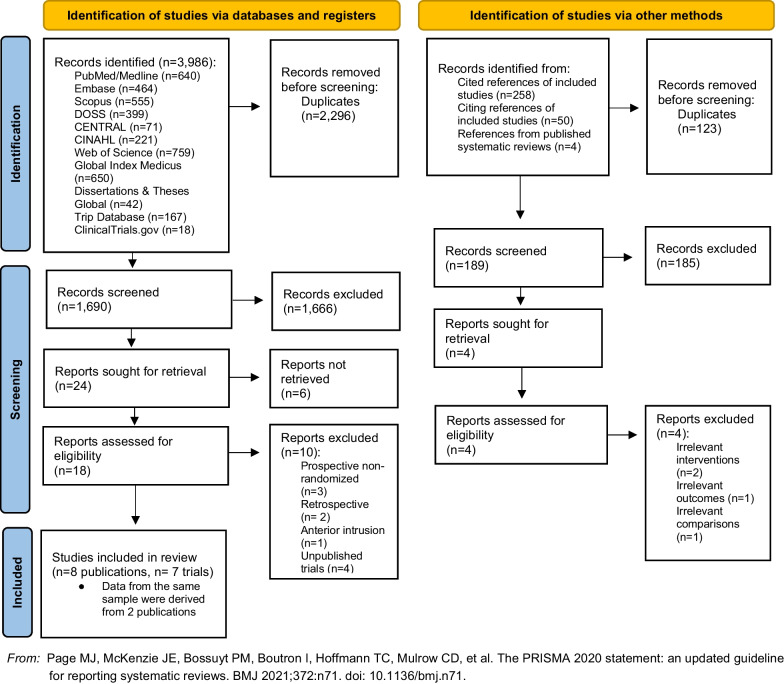
PRISMA flowchart of the included studies

A total of 3986 records were identified through the electronic data search (Fig. 1). After removal of duplicates using ProQuest RefWorks (Ann Arbor, USA) reference management software, 1690 articles remained for screening. A total of 1666 articles were excluded following titles and abstracts screening, and 24 reports were sought for retrieval. Subsequently, 18 were assessed for eligibility, of which 10 were excluded (Appendix 2). Finally, 8 articles [25–32], 2 of which reported data from the same trial [26, 27] were included in this review.

### Study characteristics

Table 2 summarizes the characteristics of the included randomized controlled trials. All the included trials were 2-arm parallel RCTs [25–28, 30–32], except one study that was three-arm parallel group RCT [29]. All of the included trials were conducted in a university setting in various countries: Brazil [31], India [28], Egypt [26, 27], Syria [29, 30, 32] and Spain [25]. The identified studies included a total of 242 subjects. The age range fell between 7 and 40 years. Duration of the active phase of intrusion ranged between 6 months [25], 8–10 months [28, 29, 32], and 12 months [30, 31]. One study only reported a retention phase of 10 months [28].

**Table 2:** Characteristics of the identified trials (*n* = 7)

Study and setting	Participants and treatment groups	Inclusion criteria	Appliance used/design/force	Prescribed wear time	Outcome assessment/follow up/duration of intrusion	Outcomes
Torres et al. [31] (2006) University setting—Brazil	***n* = **60 **E (crib and highpull chin cup): *n* = **30 (22F, 8M); 8.3 y (7–10.1 y) **C (no treatment):** *n* = 30 (23F, 7M); 8.6 y (6.8–10.4 y)	(6–10 y) Class I AOB Non-extraction No missing teeth or cross bite Absence of oral habits and airway obstruction	**Removable palatal crib:** Adams clasps U6’s, labial bow, palatal crib, acrylic coverage High pull chin cup: **450–550 g/side**	14–16 h for 12 m	Pre- and post-TT lateral cephalograms T 2– T 1 changes over time 34 skeletal, dentoalveolar and Soft tissue measurements **Duration of intrusion: 12 m**	**Skeletal** SNA (°), SNB (°), ANB (°), ALFH (mm), SN. GoGn (°) **Dentoalveolar** U6 Eruption (mm), L6 Eruption (mm), difference in eruption of molar (mm), overbite (mm), L6-GoMe (mm), L1-GoMe (mm), U6-PP (mm), U1-PP (mm), U1.NA (°),U1-NA (mm), L1.NB (°), L1-NB (mm), U6-FHp (mm), L6-FHp (mm), U1-FHp (mm), L1-FHp (mm), Expo U1 (mm), interincisor (°) **Soft tissue** H. NB (°),Nasolabial (°) mentolabial (°), Sn-ES (mm), Gl.Sn.P ′ (°), Sn-Gl vertical (mm), P ′ -Gl vertical (mm), LS-P ′ Sn (mm), LI-P ′ Sn (mm), soft AFH (mm), interlabial gap (mm)
Doshi et al. [28] (2010) University setting—India	***n*** = 20 (12F, 8 M); (8–15 y) **E1 (spring loaded bite block):** *n* = 10 (5F, 5M) **E2 (magnetic bite block): *n* = **10 (7F, 3 M) **C:** matched data from growth study (Human Growth Research Center, University of Montreal, Quebec)	AOB Steep mandibular plane Increased gonial angle Increased lower anterior facial height Class I or II No finger-sucking habits or evidence of enlarged tonsils	**Spring loaded Bite Block:** Woodside and Linder-Aronson mandibular plate with occlusal acrylic resin block connected by buccal and lingual helical spring (0.9 mm SS), soldered to Adams clasps (0.8 mm SS) Two, a 0.9-mm SS hook were placed buccally into the occlusal bite block in the molar region to measure the amount of activation Activation: **250–300 cN/4 weeks** **Magnetic Bite block:** Active vertical corrector (Dellinger) Similar bite blocks to spring loaded, in each arch two circular (1.5*10.0 mm) neodymium iron boron magnets Force applied to teeth: **300 cN**	Full time wear	Pre- and post-TT lateral cephalograms T2–T1 changes over time 34 (18 linear and 16 angular) measurements, superimpositions EMG activity of the masseter and temporalis muscles **Duration of intrusion: 8 m** **Retention:** 10 m with passive block	**Skeletal angular** SNA, SNB, ANB, Beta **Vertical angular** SN-GoGn, Ar-Go-Me, Ar-Go-N N-Go-Me, Gn/FH, SN/ANS-PNS, SN/UOP, SN/LOP, S-Ar-Go, SN/Go-Ar **Vertical linear** SN-ANS, SN-PNS, S-Go, N-Me, S-Go/N-Me **Dentoalveolar** UI-NA, LI-NB, UIE-NA LIE-NB,UIE-ANS-PNS, LIE-Go-Gn, UMC-ANS-PNS, LMC-Go-Gn, overjet, overbite **Soft tissue (mm)** Upper lip-S line, lower lip-S line **EMG** Mean changes in masseter and temporalis activity
Akl et al. [26, 27] (2020,2021) University setting-Egypt	***n*** = 20 (8–15 y) **C (200 g intrusive force with miniscrews): *n* = **10 (19.22 ± 1.45 y) **E (400 g intrusive force with miniscrews):** *n* = 10 (18.95 ± 1.77 y)	Adult aged 18 to 25 y Skeletal open bite Dental open bite (3 to 8 mm) Skeletal Class I/mild to moderate skeletal Class II Normal incisal show Non-extraction No previous orthodontic treatment	**Pre-intrusion**: segmented fixed appliance on mx posterior teeth, Roth prescription Intrusion phase: **4 mini-screws (10 × 1.6 mm; 3 M Unitek TAD)**: 2 infrazygomatic and 2 palatal between the 1st and 2nd M Palatal wires and lower buccal stabilizing wires (0.9-mm wire bonded on the buccal surface) closed nickel–titanium (Ni–Ti) coil springs **C: 200 g, E: 400 g** Recall: 2 weeks	–	Pre- and post-TT CBCT images CBCT: Mx teeth intrusion, tipping of intruded teeth mesiodistally and buccolingually, lower teeth extrusion, root resorption: root lengths (cusp tip to the root apex of each root of all posterior teeth) Clinically: open bite closure/2 weeks **Duration of intrusion: 6 m**	Intrusion measurements (mm) to FH for U4, U5, U6, U7 Mesio-distal tipping (°) relative to Frankfort Horizontal Plane (Tooth/FH) for U4, U5, U6, U7 Change in torque relative to mid-sagittal Plane (°) (Tooth/MSP) for U4, U5, U6, U7 Root length (mm) (1st and 2nd pm: B,P), (1st and 2nd M: MB,DB,P) Open bite closure/2 weeks (clinically)
Hasan et al. [29] (2022) University setting-Syria	***n*** = 42 (26F, 16M); (8–10 y) **E1 (FPBB and LLLT):** *n* = 14 (9F, 5M); (8.97 ± 0.58 y) **E2 (FPBB):** *n* = 14 (8F, 6M); (9.02 ± 0.51 y) **C:** (no TT): *n* = 14 (9F, 5M); (9.87 ± 0.44 y)	Clinical: 8–10 y Fully erupted incisors Class I or Class II skeletal 1 mm AOB minimum No previous orthodontic tt lateral cephalograms: (SN/Go Me) angle > 34°, MM-angle > 30°, and Björk sum > 40°	**Two posterior acrylic bite blocks** (at least 2 mm thickness), connected by TPA (4 mm away from palatal mucosa) **Tongue crib** (0.9 mm SS wire) **Force: 250 g** **Laser:** Gallium aluminum arsenide (Ga-Al-As) laser with a continuous wavelength of 808 nm Laser parameters: The power of 250 mW, the energy at 4 J, application time 16 s per point Frequency of application: day 1, 3, 7, and 14 of the first month, then every 15 days until the end of the treatment	Full time wear	Pre- and post-TT lateral cephalograms Recall: monthly End of active TT: positive overbite 1–2 mm Retention: posterior bite block and crib for 10 m **Duration of intrusion:** **E1: (7.07 ± 1.54 m)** **E2: (9.42 ± 2.31 m)**	Clinical: Time to correct AOB Cephalometric: Angular: SNA°, SNB°, ANB°, SN/MP°, PP-MP (MM)°, Björk Sum°, Y-axis°, U1/SN°, L1/MP°, Linear (mm): S-Go, N-Me, S-Go/N-Me, U1-PP, U6-PP, L1-MP, L6-MP, overbite, overjet
Abellan et al. [25] (2021) University setting-Spain	***n*** = 20 (12F, 8M); (44.9 ± 9.6 y) **E (PBM and TAD based mx molar intrusion)**: *n* = 10 **C (TAD based mx molar intrusion):** *n* = 10	Extruded U6s without curved roots Good oral hygiene Permanent dentition with presence of adjacent teeth to the molar to be intruded No previous trauma, orthodontic treatment or periodontal disease Alveolar bone loss < 30% No severe crowding in posterior teeth	**Intrusion:** 2 mini-screws (Jeil Corp, Seoul, Korea) 1.6 mm × 10 mm placed buccal mesial and palatal distal to the over erupted molar Buttons were bonded buccal and lingual to molars Elastic chains **(75 g force)** between the buttons and mini-screw 0.01 SS ligature tie between miniscews and molar after intrusion is completed **PBM:** A low-power diode laser (Periowave; Ondine BioPharma, Vancouver,Canada) wavelength of 670 nm, with a power of 150 mW	–	Pre- and post-TT CBCT images At days 0 and 180 Superimposed images using Dolphin software 3D models (STL) format obtained at 0, 90, and 180 days On days 0, 1, 2, 3, 4, and 7 of the beginning of the intrusion and in each monthly follow-up **Duration of intrusion: 6 m**	Periodontal parameters (probing depth and bleeding of probing) Mean intruded distance (mm), intruded velocity mm/m at 3 m and 6 m Resorption: mean molar volume at T0 and T2 (6 m)
Mousa et al. [30] (2021) University setting-Syria	***n*** = 40 (19F, 21M), (7.5–10.5 y) **E1 (OBB):** *n* = 20, (8F, 12M), (8.8 ± 1.5 y) **E2 (RPBP/C):** *n* = 20, (11F, 9M), (8.6 ± 1.1 y)	Clinical: AOB 2–5 mm 7.5 to 10.5 y Skeletal Class I or Class II Tongue thrust No airway issue or previous orthodontic TT	**RPBP/C:** Upper removable appliance with posterior bite plane (1–2 mm thickness) **OBB:** posterior acrylic bite blocks, palatal bar (1.2 mm), to guide the tongue into a more posterior position. Labial bow (0.9 mm) to achieve a competent seal	Full time wear for 12 m Retention: night time wear	Pre- and post-TT lateral cephalograms 9 angular and 8 linear measurements **Duration of intrusion:** 12 m	Skeletal Angular (°): SNA, SNB, ANB, MM, SN.GoMe, Björk Linear (mm): S-Go, N-Me Dentoalveolar Angular (°): 1U.SN,1L. GoMe,1U.1L Linear (mm): 1U-SPP, 6U-SPP,1L-GoMe, 6L-GoMe, OJ, OB
Hasan et al. [32] (2022) University setting—Syria	***n*** = 40 (19F, 21M) **E (RMI): *n* = **20, (10F, 10M), (9.7 ± 0.66y) **C (no TT): *n* = 20** (9F, 11M), (9.9 ± 0.54 y)	Clinical: 8–12 y (mixed dentition stage) Skeletal AOB Class I, II Radiographic inclusion: (SN/GoMe) > 33° MM > 27° facial axis (Y-axis) > 65°	**RMI:** TPA and lingual arch. Nickle-titanium active springs attached to the tubes of upper and lower permanent first molars Intrusion force: 800 g per side Pulpotomy and occlusal reduction on primary molars were undertaken to reestablish occlusal contact	Full time wear	Pre- and post-TT lateral cephalograms 9 m apart 20 variables **Duration of intrusion:** 9 m	Skeletal SNA, SNB, SN/GoMe, MM, Björk Sum, Y axis, S-Go, N-Men, Dentoalveolar U1-PP, U6-PP, L6-MP, U1/SN, U1/L1, OB, OJ

1M, first molar; 2M, second molar; AOB, anterior open bite; C, control; cN, centinewtons; DB, distobuccal; E, experimental; EMG, electromyography; F, females; FPBB, fixed posterior bite block; g, gram; h, hours; Go, gonion; J, joules; kv, kilovolts; L, lower; LLLT, low-level laser therapy; M, males; m, months; MB, mesiobuccal; Me, menton; MM, maxillary mandibular angle; mm, millimeters; MP, mandibular plane; mW, milliwatts; Mx, maxillary; N, nasion; nm, nanometers; OB, overbite; OBB, open bite Bionator; OJ, overjet; P, palatal, PBM, photobiomodulation; PM, premolar; RMI, rapid molar intruder; RPBB, removable posterior bite block; RPBP, removable posterior bite plane; S, sella; SS, stainless steel; TAD, temporary anchorage device; TPA, transpalatal arch; TT, treatment; U, upper; y, years

Different appliances were utilized for intrusion, the removable palatal crib with high pull headgear was used for open bite treatment in one trial [31]. The posterior bite blocks were utilized in three trials [28–30], one compared the effects of the spring loaded to the magnetic bite blocks [28]. One study compared the effects of an upper fixed posterior bite block fixed posterior bite block (FPBB) to a no treatment group, further utilized low-level laser therapy (LLLT) in a third group of FPBB to evaluate its effect on accelerating tooth movement [29]. The removable posterior bite plane (RPBB) was used with a crib and compared to an open bite Bionator (OBB) functional appliance [30]. Additionally, the use of the rapid molar intruder (RMI) spring appliance was compared to a no treatment control in one trial [32]. As for the TAD-based maxillary molar intrusion studies, one trial compared the use of two different force magnitudes (200 g and 400 g) on the efficacy of intrusion with mini-screws [26, 27]. While the other study focused on pre-prosthetic intrusion of supraerupted molars with mini-screws, additionally assessed the effects of photobiomodulation on accelerating the rate of intrusion [25]. The 5 clinical trials that investigated the efficacy of using appliances other than TADs in intruding teeth, included subjects of a young age group [28–32], while the two studies investigating the efficacy of TADs in intrusion included adults [25, 26].

With respect to forces applied and wear instructions, the removable palatal crib with high pull headgear were prescribed for 14–16 h, with the headgear applying a force of 450–550 g/side [31]. The spring-loaded bite block was activated every 4 weeks and applied a force around 300–400 g, similar to that applied by the magnetic bite block [28]. The fixed posterior bite blocks exerted an intrusive force of approximately 250 g. The RMI exerted 800 g intrusive force on each side which gradually subsided to 450 g at the end of the first week and 250 g at the end of the second week. For the minis-screw studies, an elastomeric chain exerting a force of 75 g between each button (buccal and lingual) and 2 mini-screws (buccal and lingual) was used in Abellan et al. study [25]. While, Akl et al. compared the efficacy of 2 different force magnitudes on intrusion (200 and 400 g) [26, 27].

The duration of intrusion in the included studies ranged between 6 and 12 months. Outcome assessments were carried out with the use of lateral cephalograms [28–32], cone-beam computed tomography (CBCT) images [25–27] and 3D digital models [25].

### Risk of bias within the studies and quality assessment

Risk of bias for the included studies is presented in Table 3. Six of the included trials were judged to have some concerns mainly due to selective reporting of the results [25, 28, 30–32] and lack of information regarding blinding of outcome assessor [25, 29–32]. One trial was judged at high risk of bias due to concerns related to the randomization process and measurement of the outcomes [28]. The overall quality of evidence for the primary outcomes (the amount and rate of intrusion of the maxillary molars), assessed by GRADE (Grading of Recommendations, Assessment, Development, and Evaluations) [33] was very low due to the heterogeneity in the interventions assessed, relatively small sample size in 5 trials [25–28, 30, 32], and based on the overall assessment of the risk of bias (Table 4).

**Table 3:**
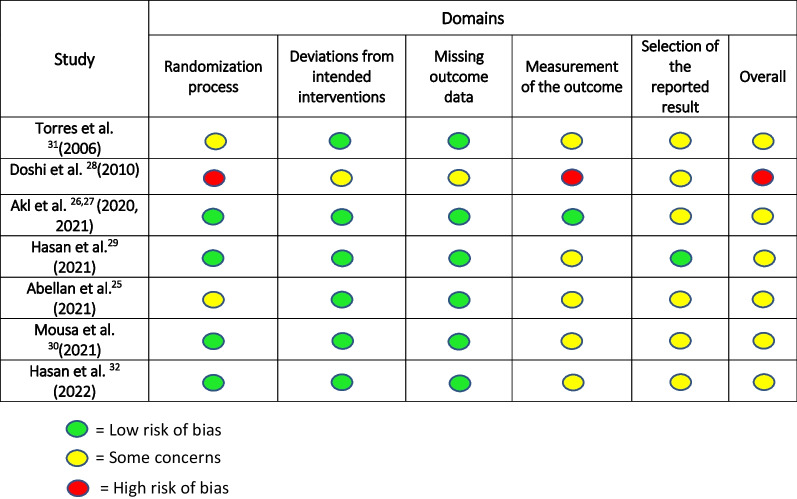
Risk of bias assessment for the included studies

**Table 4:** Certainty of available evidence for the amount and rate of maxillary molar intrusion

Quality assessment							Overall quality of evidence
No of studies and participants	Design	Risk of bias	Inconsistency	Indirectness	Imprecision	Other	
Outcomes: amount and rate of maxillary molar intrusion							
7 trials *n* = 242 participants	Randomized controlled trials	Very serious^a^	Very serious^b^	Not serious	Serious^c^	None	Very low

^a^Based on the risk of bias assessment

^b^Due to heterogeneity in interventions assessed

^c^Relatively small sample size in 5 trials

### Results of individual studies

Tables 5 and 6 summarize the primary (amount and rate of maxillary molar intrusion) and secondary (root resorption, subsequent vertical movement of lower molars, stability, patient reported outcomes, compliance, cost effectiveness and adverse events) outcomes reported in the identified trials.

**Table 5:** Findings related to the amount and rate of maxillary molar intrusion

Study and treatment groups	Amount of maxillary molar intrusion (mean (mm) ± SD)			Calculated rate of maxillary molar intrusion (mean (mm)/month ± SD)		
	*E*1	*E*2	*C*	*E*1	*E*2	*C*
Torres et al. [31] (2006) E1 (crib and HP chin cup): *n* = 30 C (no treatment): *n* = 30	0.88 ± 1.55*	–	0.26 ± 1.13	0.07 ± 0.13	–	0.02 ± 0.09
Doshi et al. [28] (2010) E1(spring-loaded bite block): *n* = 10 E2 (magnetic bite block): *n* = 10	− 0.8 ± 0.3	− 1.1 ± 0.4	–	− 0.1 ± 0.04	− 0.14 ± 0.05	–
Akl et al. [26, 27] (2020) E1 (400 g force): *n* = 10 E2 (200 g force): *n* = 10	− 2.37 ± 1.3	− 2.61 ± 1	–	− 0.4 ± 0.22	− 0.44 ± 0.16	–
Hasan et al. [29] (2021) E1 (FPBB and LLLT): *n* = 14 E2 (FPBB): *n* = 14 C (no treatment): *n* = 14	− 1.21 ± 0.32	− 0.82 ± 0.37	0.32 ± 0.37	− 0.17 ± 0.05	− 0.09 ± 0.04	–
Abellan et al. [25] (2021) E1 (PBM and TAD based mx molar intrusion): *n* = 10 E2 (TAD based mx molar intrusion): *n* = 10	− 2.31 ± 0.65	− 2.95 ± 1.16	–	− 0.42 ± 0.13	− 0.49 ± 0.17	–
Mousa et al. [30] (2021) E1 (OBB): *n* = 20 E2 (RPBP/crib): *n* = 20	− 1.44 ± 0.6	− 1.11 ± 0.8	–	− 0.12 ± 0.05	− 0.09 ± 0.07	–
Hasan et al. [32] (2022) E (RMI): *n* = 20C (no treatment): *n* = 20	− 2.90 ± 1.66	–	0.55 ± 1.93	− 0.32 ± 0.18	–	0.06 ± 0.21

C, control; E, experimental; FPBB, fixed posterior bite plane; HP, high pull; mm, millimeter; OBB, open bite Bionator; PBM, photobiomodulation; RMI, rapid molar intruder; RPBP, removable posterior bite block; SD, standard deviation; TAD, temporary anchorage device; mx, maxillary; LLLT, low-level laser therapy

*Positive value indicating molar extrusion

**Table 6:** Additional findings from the included studies

Study	Root resorption			Amount of vertical movement of mandibular molars (mean (mm) ± SD)			Stability of intrusion	Patient reported outcomes	Compliance	Cost effective-ness	Adverse events
	Parameter	*E*1	*E*2	*E*1	*E*2	*C*					
Akl et al. [26, 27] (2020,2021) E1: TADs 200 g E2: TADs 400 g	Change in length of molar roots (mean ± SD)	− 0.8 ± 0.88 mm	− 0.82 ± 0.93 mm	1.06 ± 2.06 mm	0.65 ± 0.52 mm	-	NA	NA	NA	NA	Soft tissue overgrowth around the heads of the infrazygomatic TADs TAD failure (loose): *n* = 2 (one infrazygomatic and one palatal TAD) No inter-group comparison
Abellan et al. [25] (2021) E1: PBM and TADs E2: TADs	Change in molar volume (mean ± SD)	− 52 ± 142.22 mm^3^	− 22.4 ± 160.59 mm^3^	NA	NA	NA	NA	NA	NA	NA	TAD failure: *n* = 4 (one developed soft tissue hypertrophy, and the other 3 became loose)
Torres et al. [31] (2006) E1: crib and HP chin cup C: no treatment	NA	NA	NA	1.06 ± 1.31	-	0.84 ± 1.03	NA	NA	NA	NA	NI
Doshi et al. [28] (2010) E1: spring-loaded bite block E2: magnetic bite block	NA	NA	NA	− 0.2 ± 0.3	− 0.8 ± 0.4	-	NA	NA	NA	NA	E1: breakage (*n* = 7)
Hasan et al. [32] (2021) E1: FPBB and LLLT E2: FPBB C: no treatment	NA	NA	NA	0.04 ± 0.41	0.14 ± 0.23	-	NA	NA	NA	NA	No observed harms
Mousa et al. [30] (2021) E1: OBB E2: RPBP/C	NA	NA	NA	0.19 ± 1.1	− 0.27 ± 1.3	-	NA	NA	NA	NA	E1: tongue ulceration (*n* = 2) E2: tongue ulceration (*n* = 2) and soft tissue irritation (*n* = 2)
Hasan et al. [32] (2022) E: RMI C: no treatment	NA	NA	NA	− 1.54 ± 2.18	-	0.56 ± 1.77	NA	NA	NA	NA	Spring fracture (*n* = 2)

C, control; E, experimental; FPBM, fixed posterior bite block; HP, high-pull; LLLT, low-level laser treatment; NA, not assessed; NI, no information; OBB, open bite bionator; PBM, photobiomodulation; RMI, rapid molar intruder; RPBP, removable posterior bite plane; SD, standard deviation; TAD, temporary anchorage device

### Amount of maxillary molar intrusion

With the use of a crib and high-pull (HP) chin cup, Torres et al. [31] reported no real or relative intrusion of maxillary molars, but rather upper molars were slightly extruded (0.88 ± 1.55 mm) compared to controls (0.26 ± 1.13 mm) (Table 5). Doshi et al. [28] showed that both bite blocks (spring loaded and magnetic) resulted in intrusion of maxillary molars, with more statistically significant amount of intrusion reported with the use of the magnetic bite blocks (1.1 ± 0.4* mm, *P* < 0.05) compared to the spring-loaded. Regarding the efficacy for the fixed posterior bite planes FPBB in intruding the maxillary molars, Hasan et al. [29] showed a statistically significant amount of intrusion with the use of the FPBB (− 0.82 ± 0.37 mm, P < 0.001) for 9 months compared to their control group. With the administration of LLLT, the reported amount of maxillary molar intrusion was − 1.21 ± 0.32 mm, *P* < 0.001 at 7-month follow-up. Likewise, the RMI demonstrated an active intrusion for the maxillary molars of − 2.90 ± 1.66 mm compared to the control [32]. Mousa et al. [30] compared the OBB appliance to the removable posterior bite plane RPBB with a crib, reporting almost a similar amount of maxillary molar intrusion observed with the use of both (OBB: − 1.44 ± 0.6 mm, RPBB and crib: − 1.11 ± 0.8 mm).

Regarding intrusion with TADs, Akl et al. [26, 27] showed that the amount of intrusion with either 200 g or 400 g of force (200 g: − 2.61 ± 1 mm, *P* < 0.05, 400 g: − 2.37 ± 1.3 mm, *P* < 0.05), were non-statistically significant between the studied groups at 12-month follow-up. On the other hand, Abellan et al. [25] reported an amount of intrusion of (− 2.95 ± 1.16 mm) with the use of TADs for 6 months, this amount was less with the use of photobiomodulation PBM (− 2.31 ± 0.65 mm).

### Rate of maxillary molar intrusion

The monthly rate of maxillary molar intrusion was − 0.44 ± 0.16 mm/month with the use of 200 g force with TADs [26, 27] and − 0.49 ± 0.17 mm/month when TADs were used for intrusion without PBM [25] (Table 5). The rate of molar intrusion with the use of RPBP was (− 0.09 ± 0.07 mm/month) [30] and with the use of FPBB was (− 0.09 ± 0.04 mm/month) [29] (Table 5).

### Secondary outcomes

#### Root resorption

Two of the included trials quantified and reported root resorption following intrusion with the use of TADs [25–27] (Table 6). One trial found minimal difference in terms of length of maxillary molar roots following the use of two different force magnitudes (200 g and 400 g) [26, 27]. In the other study, greater changes in the volume of maxillary molar root were observed when TADs were combined with PBM for maxillary molar intrusion, than with the former alone [25].

#### Amount of subsequent vertical movement of mandibular molars

Five studies reported the amount of subsequent vertical movement of the lower molars [26–31] (Table 6). Extrusion of the lower molars, following intrusion of its opposing, was observed with the use of the crib and high pull chin cup (1.06 ± 1.31 mm) [31], and maxillary TADs (200 g: 1.06 ± 2.06 mm, 400 g: 0.65 ± 0.52 mm) [26, 27]. Vertical control of the lower molars was reported with the use of posterior bite planes in general [28–30]; the magnetic bite block slightly intruded the lower molars (–0.8 ± 0.4 mm) [28].Likewise, the RPBB had minimal intrusive effects on the lower molars (− 0.27 ± 1.3 mm) [30]. On the other hand, the use of RMI spring appliance intruded the lower molars by (− 1.54 ± 2.18 mm) [32].

### Other secondary outcomes

None of the included trials reported outcomes related to stability of intrusion, patient reported outcomes, compliance or cost effectiveness. In terms of adverse effects, one trial reported soft tissue overgrowth around the heads of TADs, and failure of 2 miniscrews during the experiment [26, 27] (Table 6). Another trial disclosed the failure of 4 miniscrews during the experiment [25]. Breakages of the posterior bite blocks was reported for 7 subjects in Doshi et al. trial [28].Spring fractures for the RMI were observed in 2 cases [32]. Finally, Mousa et al. [30] mentioned that tongue ulcerations and soft tissue irritation were observed in some cases with the use of OBB and crib with RPBB.

#### Synthesis of the result

A meta-analysis was precluded due to the heterogeneity in interventions, population examined and outcome variables. 

## Discussion

Based on the findings of the current systematic review, there is limited and very low level of evidence concerning the effectiveness and stability of maxillary molar intrusion achieved using various mechanotherapies. While some appliances offered better vertical control of lower molar movement or even minimally intruded the lower molars, the clinical significance is questionable. Patient-reported outcomes, levels of compliance and cost-effectiveness were overlooked in the identified trials. Furthermore, no studies included long-term evaluation of stability of molar intrusion. Therefore, no recommendation can be made in favor of conventional or TAD-based treatment.

Treatment modalities assessed in the included studies varied between different age groups and malocclusions. Conventional methods of intrusion were mainly tested in a young age group [28–31], while TADS were experimented on adults [25, 26]. As a result, no studies involving a comparison of conventional methods and TADs were identified since TADs are not typically used in very young patients [34]. Treatment modalities evaluated in the identified studies were mainly focused on improving an anterior open bite by maxillary molar intrusion [26–31]. Only one RCT investigated the efficacy of TADs in intruding supra-erupted maxillary molars [25]. Overall, trials included were heterogenous in nature due to the variety of interventions, outcomes and population examined, therefore, meta-analysis was precluded.

One of the early treatment modalities that provides vertical control in open bite cases is the vertical-pull chin cup [31, 35, 36]. Majourau and Nanda found that its use with expanders prevented an increase in the anterior facial height and the mandibular plane angle [37]. Ritucci and Nanda reported that the primary effect of the chin cup is on maxillary incisors [38]. However, when used in conjunction with bite blocks it significantly intruded the upper and lower molars [39], most probably due to the effect of the bite blocks rather than the chin cup itself. Moreover, anterior extrusion is often limited to dentoalveolar changes and associated with higher relapse rate. Therefore, molar intrusion can be considered a suitable way for non-surgical correction of anterior open bites [40]. However, patient compliance is known to be problematic especially with extraoral appliances [41]. Therefore, availability of intraoral appliances that are effective in achieving intrusion of maxillary molars might offer a better alternative.

With regard to the posterior bite blocks, they come in different forms and designs and have been continuously modified (spring loaded, magnetic, fixed or removable) [9, 42–44]. They hinge the mandible open, therefore, stretching the surrounding musculature, along with the continues biting force, subsequently they would apply intrusive force on the posterior teeth, which allows the forward and upward autorotation of the mandible at a later stage for open bite closure [42, 43]. Moreover, they have been used to intrude supra erupted molars in adult subjects [45]. Three of the included RCTs evaluated the efficacy of these blocks in correcting open bite malocclusion in young age group [28–30]. It is evident that the amount of intrusion reported with the use of these bite blocks is greater than that achieved with other appliances (OBB and chin cup) [30, 31]. Moreover, integration of repelling magnets (the active vertical corrector) in these blocks seems to provide better vertical control [28]. The presence of magnets transforms the conventional acrylic blocks into energized blocks, the repelling forces of the opposing magnets constitutes the built-in energy system. Thus, providing reciprocal intrusive forces on the maxillary and mandibular teeth [8]. The active vertical corrector was found to be more effective in maxillary molar intrusion even in adults, since these magnets often generate a force that ranges between 600 and 650 g on the posterior teeth [8, 45]. Overall, studies included in the current review showed that intrusion of maxillary molars is possibly achievable with posterior bite blocks, with additional advantage of vertical control of mandibular molars and possible minor intrusion. Similar intrusion effect was observed in functional appliances that have integrated posterior bite blocks (e.g. OBB) [30]. Therefore, the use of posterior bite blocks might be a feasible option in intruding posterior teeth. Further, the integration of magnets can improve the predictability of intrusion with bite blocks [8]. A recently published study assessed the effectiveness of RMI appliance which consists of a nickel-titanium spring (RMI®, American Orthodontics, Sheboygan, USA) loaded fixed appliance. The spring extends from the upper to the lower first molar bands with metal pins, and exerts intrusive forces that is applied to the upper and lower molars. Therefore, significant amount of intrusion was reported with its use [32].

Despite the widespread use of TADs as well as skeletal anchorage devices, none of the identified studies involved a comparison of conventional and TAD-based treatments for intrusion of molars. Interestingly, the conjunctive use of TADs in the zygomatic buttress area with maxillary occlusal splint resulted in maxillary molar intrusion (2-4 mm) in 60% of the patients, while one patient had greater than 4 mm intrusion [46]. Furthermore, more predictable amount of intrusion can also be achieved with the use of miniplates (skeletal anchorage system), since they can be placed further away from the roots and provide more vertical component for the intrusive force [47, 48]. However, their limited insertion sites, cost and the surgical procedures required for their placement and removal makes them less popular among clinicians [49].

Tooth movement is a biological response to the applied orthodontic forces. The reported average rate of orthodontic tooth movement in a sagittal direction with continuous force is 0.8 to 1.2 mm/month [50]. The reported calculated rate for intrusion (vertical movement) in this review ranged between (− 0.11 ± 0.06 mm/month) for posterior bite blocks and (− 0.46 ± 0.16 mm/month) with the use of mini-screws. It seems like the rate of intrusion is less than the average rate of sagittal tooth movement. The area of intrusive force is concentrated over a small area at the apex, pushing against dense cortical bone, the distribution of bone density along the axis of the tooth differs, the coronal part of the root often moves against cancellous bone, while the apical part in intrusion is pushing against the dense cortical bone [51]. In a previous investigation, it has been shown that factors that limit the rate of tooth movement include bone density, turnover, and the degree of hyalinization in the periodontal ligament [52].

In terms of root resorption associated with intrusion, shortening of the maxillary molars’ roots [26, 27] and reduction in their volume [25] were observed in the two studies involving molar intrusion using TADs. The apex of the tooth often experiences greater amount of stress and compression during intrusion [53]. A recent systematic review has concluded that the average orthodontic induced root resorption (OIRR) following intrusion is 0.41 mm in the maxillary molars and the amount of force applied is not correlated with the amount of resorption [54]. Therefore, root resorption is an expected sequel of intrusion and difference in the severity of root resorption is expected with the use of different appliances due to variation in the force levels and duration exerted [53, 55].

Finally, the current review highlighted the amount of subsequent vertical movement of mandibular molars reported in the included trials. Significant amount of extrusion of mandibular molars was seen in Akl et al. with the use of maxillary TADs [26, 27] and in Torres et al. with the vertical-pull chin cup [31]. While, control of vertical movement of the lower molars was reported with the use of posterior bite blocks [28–30]. Additionally, an active intrusive force was applied with the RMI to the upper and lower molars in young patients [32]. Specifically, intrusion of lower molars was seen the most with the use of RMI (− 1.54 ± 2.18 mm) followed by the use of the magnetic bite blocks (− 0.8 ± 0.4 mm) and to a lesser extent with the springloaded bite block (− 0.2 ± 0.3 mm) [28], and OBB (− 0.27 ± 1.3 mm) [30]. Some authors suggested ligation of the lower molars to a mandibular mini-screw during intrusion [46]. This denotes the importance of controlling the vertical movement of lower molars while intruding the uppers, especially for overbite correction.

### Strengths and limitations

In the current systematic review, overall quality of evidence ranged from low to very low due to methodological shortcomings observed in the included RCTs. Moreover, conduction of meta-analysis was precluded due to the insufficient homogeneity between the studies. This is typical in orthodontic systematic reviews, with more than two thirds are lacking a meta-analysis [56]. It is clear that that there is a need for further high-quality research encompassing outcomes related to efficacy and stability of the different interventions in terms of the amount and rate of intrusion; as well as cost-effectiveness, compliance levels, and patient-reported outcomes.

All the included trials were single-centered and undertaken in university hospitals. This was previously highlighted, in which just less than 15% of orthodontic trials published over a period of 5 years being practice-based and multi-centered [57]. Moreover, the observation period for the included trials was relatively confined to the treatment duration without retention or follow-up period, therefore the amount of relapse following intrusion was not considered.

The current systematic review comes as the second review following the one published in 2006 by NJ et al. [20] to quantify the amount and rate of maxillary molar intrusion. Over the last 15 years, the use of TADs became common and their effectiveness in intrusion was not assessed in any previous analysis. Moreover, CBCT scans would allow better assessment for the amount of intrusion, but with their limited use due to radiation concerns, majority of the included studies used 2D cephalometric radiographs to evaluate the amount of intrusion.

### Implications for research and clinical practice

Future RCTs should be considered to further our knowledge on the efficacy of different interventions (conventional vs TADs or a combination between both: TADs and miniplates) in intrusion. These trials should focus on the amount and rate of posterior molar intrusion and use the up-to-date 3D radiographs for outcome assessment. Furthermore, they should take into consideration an observation period for relapse assessment and evaluate patient reported outcomes.

## Conclusions

There is limited evidence related to the effectiveness of different appliances in achieving maxillary molar intrusion. The use of temporary anchorage devices seems to be clinically efficient in maxillary molar intrusion despite their frequent reported failure. Some of these appliances (such as spring loaded or magnetic posterior bite blocks) and the RMI provide posterior occlusal coverage, therefore, offer the additional advantage of intruding the mandibular molars. However, long-term stability of the amount of intrusion achieved is unclear. The findings were based on individual studies with small sample size. Patient-reported outcomes, compliance levels, cost-effectiveness and long-term stability were not assessed in any of the included studies. Limited number of studies assessed molar intrusion for pre-prosthetic management. The level of evidence found was of very low quality. Therefore, future well-designed clinical trials should quantify the true amount of molar intrusion achieved using various interventions.

## Data Availability

The data underlying this article are available in the article and in its online material.

## Appendix 1: search strategy (from inception to March 22nd, 2023)

**Table:**

Database	Search strategy	Hits
PubMed/Medline (including Pre-Medline and non-Medline)	("Orthodontics"[Mesh] OR orthodont*[tw] OR "Open Bite"[Mesh] OR "open bite"[tw]) AND ("Molar"[Mesh] OR molar*[tw] OR "posterior teeth"[tw]) AND (intrus*[tw] OR intrud*[tw]) NOT ("Animals"[Mesh] NOT ("Humans"[Mesh] AND "Animals"[Mesh]))	640
Embase (Elsevier)	('orthodontics'/exp OR orthodont*:ti,ab,kw OR "open bite":ti,ab,kw) AND ('molar tooth'/exp OR molar*:ti,ab,kw) AND ('intrusion'/exp OR intrus*:ti,ab,kw OR intrud*:ti,ab,kw) NOT ('animal'/exp NOT ('human'/exp AND 'animal'/exp))	464
Scopus (Elsevier)	TITLE-ABS-KEY((orthodont* OR "open bite") AND (molar* OR "posterior teeth") AND (intrus* OR intrud*)) AND (LIMIT-TO (EXACTKEYWORD, "Human") OR LIMIT-TO(EXACTKEYWORD, "Humans"))	555
Dentistry & Oral Science Source (Ebsco)	(DE "ORTHODONTICS" OR DE "MALOCCLUSION" OR DE "ORTHODONTIC appliances" OR TI orthodont* OR AB orthodont* OR TI "open bite" OR AB "open bite") AND (DE "MOLARS" OR TI molar* OR AB molar* OR TI "posterior teeth" OR AB "posterior teeth") AND (TI intrus* OR AB intrus* OR TI intrud* OR AB intrud*)	399
Cochrane Central Register of Controlled Trials (Wiley)	(orthodont* OR "open bite") AND (molar* OR "posterior teeth") AND (intrus* OR intrud*)	71
Cumulative Index to Nursing & Allied Health Literature (CINAHL) Plus with Full Text (Ebsco)	(MH "Orthodontics" OR TX orthodont* OR TX "open bite") AND (MH "Molar + " OR TX molar* OR TX "posterior teeth") AND (TX intrus* OR TX intrud*)	221
Web of Science (Medline, BIOSIS Previews, and the Zoological Record only) (Clarivate)	(orthodont* OR "open bite") AND (molar* OR "posterior teeth") AND (intrus* OR intrud*)	759
Global Index Medicus (World Health Organization)	(orthodont* OR "open bite") AND (molar* OR "posterior teeth") AND (intrus* OR intrud*)	650
Dissertations & Theses Global (ProQuest)	ab((orthodont* OR "open bite") AND (molar* OR "posterior teeth") AND (intrus* OR intrud*)) OR ti((orthodont* OR "open bite") AND (molar* OR "posterior teeth") AND (intrus* OR intrud*)) OR su((orthodont* OR "open bite") AND (molar* OR "posterior teeth") AND (intrus* OR intrud*)) OR diskw((orthodont* OR "open bite") AND (molar* OR "posterior teeth") AND (intrus* OR intrud*))	42
Trip Database (tripdatabase.com)	(orthodont* OR "open bite") AND (molar* OR "posterior teeth") AND (intrus* OR intrud*)	167
ClinicalTrials.gov	Condition or disease: "open bite" OR "molar intrusion"	18

## Appendix 2: excluded studies with reasons for exclusion

**Table:**

Title of study	Reason for exclusion
Al-Falahi, B., A.M. Hafez, and M. Fouda, Intrusion of Maxillary Posterior Teeth to Correct a Severe Anterior Open Bite. Journal of Clinical Orthodontics, 2017. 51(6): p. 326–334	Prospective non-randomized
Marzouk, E.S., E.M. Abdallah, and W. El-Kenany, Molar Intrusion in Open-bite Adults Using Zygomatic Miniplates. International Journal of Orthodontics, 2015. 26(2)	Prospective non-randomized
Barbre, R.E. and P.M. Sinclair, A cephalometric evaluation of anterior openbite correction with the magnetic active vertical corrector. The Angle Orthodontist, 1991. 61(2): p. 93–102	Prospective non-randomized
Ding WH, Li W, Chen F, Zhang JF, Lv Y, Chen XY, Lin WW, Fu Z, Shi JJ. Comparison of molar intrusion efficiency and bone density by CT in patients with different vertical facial morphology. Journal of oral rehabilitation. 2015 May;42(5):355–362	Retrospective
Çinsar, A., A.R. Alagha, and S. Akyalçın, Skeletal open bite correction with rapid molar intruder appliance in growing individuals. The Angle Orthodontist, 2007. 77(4): p. 632–639	Retrospective
Polat-Özsoy, Ö., et al., Comparison of the intrusive effects of miniscrews and utility arches. American Journal of Orthodontics and Dentofacial Orthopedics, 2011. 139(4): p. 526–532	Anterior intrusion
Erbay, Elif, Türköz Ugur, and Mustafa Ülgen. The effects of Frankel's function regulator (FR-4) therapy on the treatment of Angle Class I skeletal anterior open bite malocclusion. American Journal of Orthodontics and Dentofacial Orthopedics 108.1 (1995): 9–21	Irrelevant intervention
Kiliaridis, Stavros, Inger Egermark, and Birgit Thilander. Anterior open bite treatment with magnets. The European Journal of Orthodontics 12.4 (1990): 447–457	Irrelevant outcomes
Rossato PH, Fernandes TM, Urnau FD, de Castro AC, Conti F, de Almeida RR, Oltramari-Navarro PV. Dentoalveolar effects produced by different appliances on early treatment of anterior open bite: a randomized clinical trial. The Angle Orthodontist. 2018 Nov;88(6):684–691	Irrelevant comparisons
Slaviero T, Fernandes TM, Oltramari-Navarro PV, de Castro AC, Conti F, Poleti ML, de Almeida MR. Dimensional changes of dental arches produced by fixed and removable palatal cribs: A prospective, randomized, controlled study. The Angle Orthodontist. 2017 Mar;87(2):215–222	Irrelevant interventions
The effect of molar intrusion in fixed appliance with temporary anchorage devices vs Clear aligner – unpublished clinical trial	Clinical trial abstract
Molar intrusion in open bite treatment	Clinical trial abstract
Evaluation of posterior segment intrusion using miniplates in skeletal Class II hyperdivergent adolescence: A randomized control trial	Clinical trial abstract
Skeletal and dentoalveolar changes following posterior teeth intrusion with high pull headgear in the treatment of patients with anterior open bite	Clinical trial abstract

## Abbreviations

FPBB: Fixed posterior bite block
HP: High pull
LLLT: Low-level laser therapy
OBB: Open bite bionator
RMI: Rapid molar intruder
RPBB: Removable posterior bite block
RPBP: Removable posterior bite plane
TADs: Temporary anchorage devices
